# Long‐term antipsychotic polypharmacy prescribing in secondary mental health care and the risk of mortality

**DOI:** 10.1111/acps.12906

**Published:** 2018-05-29

**Authors:** G. Kadra, R. Stewart, H. Shetty, J. H. MacCabe, C.‐K. Chang, D. Taylor, R. D. Hayes

**Affiliations:** ^1^ Institute of Psychiatry, Psychology and Neuroscience King's College London London UK; ^2^ South London and Maudsley NHS Foundation Trust London UK

**Keywords:** mortality, antipsychotics

## Abstract

**Objectives:**

To investigate the association between long‐term antipsychotic polypharmacy use and mortality; and determine whether this risk varies by cause of death and antipsychotic dose.

**Methods:**

Using data from a large anonymised mental healthcare database, we identified all adult patients with serious mental illness (SMI) who had been prescribed a single antipsychotic or polypharmacy, for six or more months between 2007 and 2014. Multivariable Cox regression models were constructed, adjusting for sociodemographic, socioeconomic, clinical factors and smoking, to examine the association between APP use and the risk of death.

**Results:**

We identified 10 945 adults with SMI who had been prescribed long‐term antipsychotic monotherapy (76.9%) or APP (23.1%). Patients on long‐term APP had a small elevated risk of mortality, which was significant in some but not all models. The adjusted hazard ratios for death from natural and unnatural causes associated with APP were 1.2 (0.9–1.4, *P* = 0.111) and 1.1 (0.7–1.9, *P* = 0.619) respectively. The strengths of the associations between APP and mortality outcomes were similar after further adjusting for % BNF antipsychotic dose (*P* = 0.031) or olanzapine equivalence (*P* = 0.088).

**Conclusion:**

The findings suggest that the effect of long‐term APP on mortality is not clear‐cut, with limited evidence to indicate an association, even after controlling for the effect of dose.

**Significant outcomes**

- As compared to long‐term antipsychotic monotherapy use, there was a weak association between long‐term antipsychotic polypharmacy use with all‐cause mortality and with natural causes of death, after adjusting for a range of possibly confounders.
- There was no significant association between APP and unnatural causes of death.
- There was no evidence that antipsychotic dose had a direct effect on the risk of death in patients with serious mental illnesses.

**Limitations**

- Despite adjusting for a wide array of possible confounders, we were unable to measure factors such as duration of mental disorder, therefore it is possible that some residual confounding may have occurred.
- We were unable to examine the association of specific antipsychotic combinations with mortality.

## Introduction

Patients with serious mental illnesses (SMI) have been estimated to die approximately 15 to 20 years earlier than the general population 1, 2. The widespread prescribing of medication regimens not recommended by existing guidelines 3, 4, such as antipsychotic polypharmacy (APP), has been suggested to contribute to this increased mortality 5, 6. Furthermore, this risk has been reported to increase with high‐prescribed dose, especially when exceeding statutory recommendations 7, 8, 9. However, on closer examination, there have been considerable differences in findings between studies examining APP, whether of long‐term and unspecified duration 5, 6, 10, 11, 12. Furthermore, methodological problems, such as examining small and homogenous samples 5, 6 and failure to adjust for covariates such as dose 11, 12, have resulted in limited generalizability and possible residual confounding. Consequently, the association between APP and serious outcomes such as mortality remains unclear.

The study described here addresses a call for further research to examine the risk of outcomes such as mortality for patients prescribed regular long‐term APP 13, 14. Our aim was to determine whether there was an association between long‐term use of APP and mortality in a large clinical cohort, using a de‐identified mental health records database. Furthermore, we set out to investigate whether this risk varies by cause of death and whether it was related to antipsychotic dose. We hypothesised that patients receiving long‐term APP would be at increased risk of all‐cause mortality, in comparison with patients on long‐term monotherapy. We further expected that mortality would be greater for natural causes of death and that patients on higher combined dose would have higher mortality. In addition, we investigated whether patients on higher doses are at increased risk of death and the extent to which this accounted for any associations with APP.

## Methods

We constructed a retrospective cohort study, using anonymised data from South London and Maudsley NHS Foundation Trust (SLAM) electronic health records (EHR) between 1st January 2007 and 31st December 2014. SLAM is one of the largest providers of secondary healthcare in Europe, serving four London boroughs (Lambeth, Southwark, Lewisham and Croydon) and a population of approximately 1.36 million 15, 16. The Clinical Record Interactive Search (CRIS) system was developed in 2008 to allow researchers to search and retrieve anonymised SLAM electronic health records (EHRs). Currently, over 280 000 cases are represented in the system. CRIS was approved for secondary analysis by the Oxfordshire Research Ethics Committee C (reference 08/H606/71+5) in 2008.

Using CRIS, we identified all adults with a diagnosis of schizophrenia (ICD‐10 code: F20.x), schizoaffective disorder (F25.x) or bipolar disorder (F31.x), who were in contact with SLAM clinical services during the observation period. All‐cause mortality was determined through the exact date of death of patients that had died in the observation period. We further determined the specific cause of death for each patient through a data linkage with the Office of National Statistics mortality data, a process whereby anonymised BRC IDs are linked to the death register 16. Causes of death were categorised into two groups. Unnatural death included ICD10 diagnosis codes of death: S00‐T98 (injury, poisoning and certain other consequences of external causes); V01‐Y98 (external causes of morbidity and mortality); and U509 (death from injury or poisoning, or event awaiting determination). All other codes were classified as natural causes of death.

Antipsychotic medication data were extracted from SLAM's pharmacy‐dispensing database and from structured and free‐text fields [using a natural language processing application (NLP)] in the source health records accessed by CRIS. NLP applications and features have been used to derive a large volume of meta‐data in CRIS for previous and current research 16, 17, 18. We have described the procedure for data extraction in detail in a separate publication 17. All antipsychotic drugs listed in the British National Formulary 65 were considered. A long‐term APP episode was defined as the first record of concurrent prescription of two or more antipsychotics for six or more months, in the observation window. A detailed description of how APP was derived is provided in Kadra et al. 17. If an APP episode was not recorded, we looked for the first episode of antipsychotic monotherapy in the observation period: where a patient was prescribed a single antipsychotic for six or more months. For each patient, the follow‐up time commenced at the point they were receiving APP or monotherapy for six or more months (index date). Follow‐up continued until a death was recorded or the end of the observation period (31st December 2014), whichever occurred first.

Information on antipsychotic dose was extracted from free‐text, using natural language processing (NLP) and structured fields, for both antipsychotic monotherapy and polypharmacy, where such information was available. APP cases where dose was not available for all antipsychotics that were part of the polypharmacy were not included. The authors calculated the positive predictive value (i.e. precision) for antipsychotic dose at 0.8, in this study.

Dose was calculated at the index date using two different methods. In the UK, percentage out of maximum BNF recommended dose (%BNF) is recommended by the Royal College of Psychiatrists 19 and was calculated by converting the dose of each drug into a percentage of the BNF maximum recommended dose for that drug. For APP, the percentages for individual antipsychotics were added together into a summed value. A cumulative dose of more than 100% was considered a high dose 19. A likelihood ratio test indicated that it was appropriate to use this as a continuous variable in the analysis. In addition, we also calculated olanzapine equivalence 20 by adding up the equivalence doses of all antipsychotics that were part of the polypharmacy regimen. A total dose above 20 milligrams (mg) was classified a high dose 20, 21. A likelihood ratio test indicated that it was most appropriate to use this variable as categorical, where 1–10 mg was identified as a low dose, 11–20 mg as medium dose and 21 mg or above as high dose.

Age, gender, ethnicity and relationship status were derived from structured fields, closest to the index date. A likelihood ratio test indicated that it was appropriate to use age as a continuous variable in the analysis. Seventeen ethnic groups were collapsed into six categories due to small numbers in some cells. Relationship status was categorised as in ‘relationship’ (cohabitating, married or civil partnership) and ‘no relationship’ (single, divorced, separated, widowed, unknown). We used a neighbourhood‐level index of multiple deprivation to estimate socioeconomic status based on seven domains of deprivation ascertained from 2007 UK Census estimates (employment, income, education, health, barriers to housing and services, crime and living environment). Multiple deprivation indices were weighted and combined into an overall score applied to lower super output geographical areas (LSOAs), each containing on average 1500 residents 22. LSOAs were categorised in tertiles in the analysis. In addition, homelessness 23 was ascertained based on ‘no fixed abode’ codes.

Clinical covariates included comorbid diagnoses of depression (ICD‐10: F32, F33), personality disorder (ICD‐10: F60‐61), or substance use (ICD‐10: 10‐16), prior to or at the point follow‐up began. We ascertained this using information available from free‐text (such as progress notes) and structured fields (from drop down menus). In addition, we identified the lengths of time in days that each patient was known to SLAM services at the index date, by examining all structured and free‐text records available since 1st January 2007 up until the point the patient qualified for the APP or monotherapy group. A likelihood ratio test indicated that it was appropriate to enter this as a continuous variable in the analysis. Given the increased risk of mortality amongst smokers 24, 25, patients were classified into two groups, those who have never smoked and past or current smokers.

### Statistical analysis

stata 13 (StataCorp LP, College Station, TX, USA) was used to conduct all statistical analyses. Sample characteristics were summarised for the total cohort, as well as for all those who were in the long‐term APP and monotherapy group. Cox proportional hazard ratios were used to determine whether any of the covariates were significantly associated with all‐cause mortality. We further used chi‐square tests to investigate whether the monotherapy and APP group differed in relation to their sample characteristics. Kaplan–Meier curves with a log‐rank test were used to compare those who were prescribed APP and monotherapy in relation to all‐cause mortality. Following checks of proportional hazards assumptions, Cox regression procedures were used to examine the associations between antipsychotic regimen and risk of death.

Multivariable models included potential confounders such as age, gender, ethnicity, relationship status, deprivation status, comorbid diagnoses, time known to SLAM and smoking. Two additional fully adjusted models including %BNF and olanzapine equivalence dose, respectively, were also run. To reduce the effect of confounding by indication, we used a standard propensity score method, where the propensity score was the probability of being placed on APP based on all variables described above (apart from dose). Dose was not included in calculating the propensity score due to not having available dose information for all patients in the cohort. The propensity scores were built through a regression model, which included all covariates. We then included the propensity score as a covariate in place of all of the aforementioned confounders in the Cox model. To examine the risk for cause‐specific mortality, we used competing risk regression analyses, which allows for more than one competing risk in the cohort (e.g. different causes of death).

## Results

We identified 10 945 individuals who met the inclusion criteria for the study. The mean time of follow‐up was 1636 days (standard deviation = 839), which is approximately four and a half years. Table 1 summarises the sample characteristics by antipsychotic monotherapy and polypharmacy group. In total, 8421 (76.9%) sample cases were prescribed long‐term monotherapy, of whom 758 (9%) died in the follow‐up. A further 2524 sample cases (23%) were prescribed long‐term APP, of whom 162 (6.4%) died. Out of the patients who were prescribed long‐term APP and died, 44 (27%) were on APP just prior to their death (results not shown). Patients prescribed monotherapy differed significantly from those prescribed APP across all sociodemographic, socioeconomic, clinical and smoking characteristics apart from comorbid substance use, where a comparable proportion of patients received a comorbid substance use diagnoses. Patients prescribed APP were on average younger, more likely to be of Black African or Caribbean ethnicity, less likely to be in a relationship or employed and living in a higher deprivation neighbourhood. Furthermore, patients receiving APP were more likely to be diagnosed with schizophrenia, whereas patients on monotherapy had a higher prevalence of bipolar affective disorder diagnosis. Patients on monotherapy were more likely to have a comorbid depression diagnosis, whereas patients on APP had a higher prevalence of personality disorders. Patients receiving APP were known to the source mental health service for longer, were more likely to receive a high antipsychotic dose (as measured by both %BNF and olanzapine equivalence) and were more likely to have ever smoked.

**Table 1:** Sample characteristics of patients prescribed monotherapy and antipsychotic polypharmacy (*n* = 10 945)

Variables	Monotherapy^a^ *n* (%)	Antipsychotic polypharmacy^a^ *n* (%)
Total	8421 (76.9)	2524 (23.1)
Sociodemographic and socioeconomic factors		
Age mean (SD)	42.2 (15.4)	38.1 (13.5)
Gender		
Female	3737 (44.4)	1054 (41.8)
Male	4684 (55.6)	1470 (58.2)
Ethnicity group		
British	3160 (37.6)	838 (33.2)
Other White	791 (9.4)	184 (7.3)
Asian	566 (6.7)	159 (6.3)
Caribbean	1072 (12.7)	354 (14.0)
Black African	2198 (26.1)	813 (32.2)
Other	634 (7.5)	176 (7.0)
Employment		
Not in paid employment	8132 (96.6)	2461 (97.5)
Paid employment	289 (3.4)	63 (2.5)
Relationship status		
No relationship	7198 (85.5)	2303 (91.2)
Relationship	1223 (14.5)	221 (8.8)
Deprivation level in area of residence		
Low level	2726 (32.6)	805 (32.2)
Medium level	2758 (33.0)	808 (32.3)
High level	2742 (32.8)	821 (32.9)
Homelessness	135 (1.6)	65 (2.6)
Clinical factors		
Schizophrenia (ICD‐10: F20)	5896 (70.0)	1950 (77.3)
Schizoaffective disorder (ICD‐10: F25)	639 (7.6)	235 (9.3)
Bipolar affective disorder (ICD‐10: F31)	1886 (22.4)	339 (13.4)
Comorbid depression (ICD‐10: F32‐33)		
No	7235 (85.9)	2223 (88.1)
Yes	1186 (14.1)	301 (11.9)
Comorbid personality disorder (ICD‐10: F60‐61)		
No	7642 (90.8)	2145 (85.0)
Yes	779 (9.2)	379 (15.0)
Comorbid substance use^a^ (ICD‐10: F10‐16)		
No	7581 (90.0)	2252 (89.2)
Yes	840 (10.0)	272 (10.8)
Time known to SLAM (days)		
Mean (SD)	1603.5 (1138.2)	2223.9 (1468.9)
%BNF		
Mean (SD)	45.8 (36.8)	101.8 (68.8)
Olanzapine equivalence dose		
1–10 mg	4341 (55.7)	134 (6.0)
11–20 mg	2427 (31.2)	557 (25.0)
21 + mg	1022 (13.1)	1536 (69.0)
Smoking		
Never smoked	3016 (35.8)	374 (14.8)
Have smoked ever	5405 (64.2)	2150 (85.2)

a There was a significant difference between groups for all factors, apart from comorbid substance use (*P* = 0.242).

Table 2 describes the characteristics of the total cohort, together with an age and gender adjusted Cox regression analysis of the association between death and sample characteristics. In total, 920 (8.4%) patients died within the observation window. Age, male gender, comorbid substance use and having ever smoked were all associated with an increased risk for all‐cause mortality, whereas being in a relationship at the time the follow‐up began or being of Black African or Caribbean ethnicity was associated with a lower risk.

**Table 2:** Cox regression analysis of the association between sample characteristics and all‐cause mortality hazard (*n* = 10 945; 920 deaths)

Variables	*n* (%)	HR (95% CI)^a^
Sociodemographic and socioeconomic factors		
Age		
Mean (SD)	57.4 (17.0)	**1.1 (1.06–1.07)**
Gender		
Female	427 (46.4)	Reference
Male	493 (53.6)	**1.4 (1.2–1.6)**
Ethnicity group		
British	468 (50.9)	Reference
Other White	91 (9.9)	0.8 (0.6–1.0)
Asian	54 (5.9)	0.8 (0.6–1.0)
Caribbean	140 (15.2)	**0.7 (0.6–0.9)**
Black African	129 (14.0)	**0.8 (0.7–0.9)**
Other	38 (4.1)	0.8 (0.6–1.2)
Relationship status		
No relationship	808 (87.8)	Reference
Relationship	112 (12.2)	**0.8 (0.6–0.9)**
Employment		
Not in paid employment	910 (98.9)	Reference
Paid employment	10 (1.1)	0.6 (0.3–1.1)
Deprivation level in area of residence		
Low level	328 (35.7)	Reference
Medium level	299 (32.8)	1.0 (0.9–1.2)
High level	283 (30.9)	0.9 (0.8–1.1)
Homelessness	5 (0.6)	0.6 (0.2–1.4)
Clinical factors		
Diagnosis		
Schizophrenia (ICD‐10: F20)	690 (75.0)	Reference
Schizoaffective disorder (ICD‐10: F25)	60 (6.5)	0.9 (0.7–1.2)
Bipolar Affective Disorder (ICD‐10: F31)	170 (18.5)	0.9 (0.8–1.1)
Comorbid Depression (ICD 10: F32‐33)		
No	816 (88.7)	Reference
Yes	104 (11.3)	0.9 (0.7–1.2)
Comorbid Personality Disorder (ICD 10: F60‐61)		
No	858 (93.3)	Reference
Yes	62 (6.7)	1.2 (0.9–1.6)
Comorbid Substance Use (ICD 10: F10‐16)		
No	835 (90.8)	Reference
Yes	85 (9.2)	**1.7 (1.3–2.1)**
Time known to SLAM (days)		
Mean (SD)	1667.15 (996.7)	1.0 (0.9999–1.0000)
%BNF		
Mean (SD)	53.95 (49.7)	1.0 (0.9991–1.0017)
Olanzapine equivalence dose		
1–10 mg	373 (45.5)	Reference
11–20 mg	211 (25.7)	0.9 (0.8–1.2)
21 + mg	236 (28.8)	1.1 (0.9–1.3)
Smoking		
Never smoked	301 (32.7)	Reference
Have smoked ever	619 (67.3)	**1.5 (1.3–1.8)**

Bold indicates statistically significant value (*P* < 0.05).

a All HR have been age and gender adjusted.

**Figure 1:**
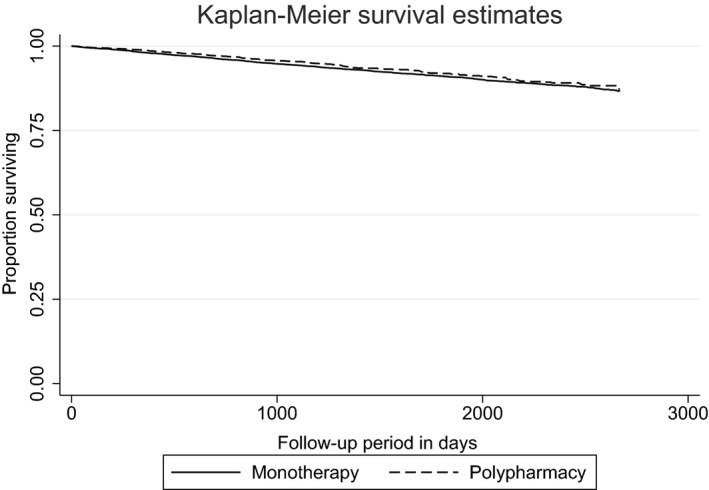
Kaplan–Meier survival curve comparing mortality over time of patients prescribed either long‐term antipsychotic monotherapy or polypharmacy (*n* = 10 945).

Figure 1 displays the Kaplan–Meier curves comparing mortality over time for patients prescribed either long‐term antipsychotic monotherapy or polypharmacy. There was no significant difference in mortality across the two groups over time (*P* = 0.166).

Table 3 summarises Cox proportional hazards models of the associations between long‐term APP (compared to long‐term monotherapy) and all‐cause mortality. Age and gender appeared to have a negative confounding effect, and adjusting for those in the multivariable model increased the strength of the association of interest. However, adjusting the model for smoking status resulted in a decrease in the hazard ratio for mortality and the association was no longer statistically significant. The fully adjusted model indicated a slightly elevated risk, but this was not statistically significant. The association remained unchanged after we adjusted for propensity scores, in place of the above factors. Antipsychotic dose information was available for 92% of the sample; therefore the total cohort sample for the analysis including %BNF dose was *n* = 10 022. Gardner et al. 26, do not provide an olanzapine equivalent dose for asenapine, therefore five further cases were dropped resulting in *n* = 10 017 for the analysis including olanzapine equivalence dose. We included %BNF and olanzapine equivalence dose as covariates in two separate models. The %BNF dose adjustment resulted in a modest significant association between APP prescribing and mortality; however, this association was not significant in the model where olanzapine equivalence was included as a covariate.

**Table 3:** Multivariate Cox regression analysis of the association between antipsychotic polypharmacy prescribing and mortality in individuals with serious mental illness. (*n* = 10 945)

Models	Antipsychotic polypharmacy vs. monotherapy	
	HR (95% CI)	*P* value
Unadjusted model	0.9 (0.7–1.1)	*P* = 0.166
Model adjusted for age and gender	**1.2 (1.0–1.5)**	***P*** ** = 0.016**
Model adjusted for socio‐demographic^a^ and socioeconomic^b^ factors	**1.2 (1.0–1.5)**	*P* ** = 0.020**
Model adjusted for age, gender and clinical factors c	**1.2 (1.0–1.5)**	*P* ** = 0.017**
Model adjusted for age, gender and smoking	1.1 (0.9–1.4)	*P* = 0.111
Fullyadjusted model^d^	1.2 (0.9–1.4)	*P* = 0.079
Fully adjusted model using propensity score as a covariate^d^	1.2 (0.9–1.4)	*P* = 0.084
Fully adjusted model and %BNF dose^e^, ^d^	**1.3 (1.0–1.5)**	***P*** ** = 0.031**
Fully adjusted model and olanzapine equivalence dose^f^, ^d^	1.2 (0.9–1.5)	*P* = 0.088

Natural causes of death	HR (95% CI)	*P* value
Unadjusted model	0.8 (0.7–1.0)	*P* = 0.062
Fully adjusted model^d^	1.2 (0.9–1.4)	*P* = 0.111
Fully adjusted model and %BNF^e^, ^d^	**1.3 (1.0–1.6)**	***P*** ** = 0.040**
Fully adjusted model and Olanzapine equivalence dose^f^, ^d^	1.2 (0.9–1.5)	*P* = 0.166

Unnatural causes of death	HR (95% CI)	*P* value
Unadjusted model	1.1 (0.7–1.8)	*P* = 0.601
Fully adjusted model^d^	1.1 (0.7–1.9)	*P* = 0.619
Fully adjusted model and %BNF^e^, ^d^	0.9 (0.6–1.7)	*P* = 0.960
Fully adjusted model and Olanzapine equivalence dose^f^, ^d^	1.1 (0.6–1.9)	*P* = 0.821

Bold indicates statistically significant value (*P* < 0.05).

a Sociodemographic factors included age, gender, ethnicity, relationship status.

b Socioeconomic factors included employment and deprivation level.

c Clinical factors comprised comorbid depression (ICD‐10: F32‐33), personality disorder (ICD‐10: F60‐61) and substance use (ICD‐10: F10‐16); and time known to SLAM services (days).

d Includes all of the above.

e Antipsychotic dose information was available for 92% of the sample; therefore the total cohort sample for the analysis including %BNF dose was *n* = 10 022.

f Gardner et al. 26, do not provide an olanzapine equivalent dose for asenapine, therefore the total cohort sample for the analysis was *n* = 10 017.

Table 3 further summarises the crude and fully adjusted competing risk regression analyses of the associations between being prescribed long‐term APP, natural and unnatural causes of death. Cause of death was available for 892 (97%) of all deaths recorded for the monotherapy and APP groups. For natural causes of death, adjusting for possible confounders indicated a modest effect of APP, which did not reach statistical significance. To examine the effect of dose, we conducted a stratification (results not shown), which indicated a significant interaction between antipsychotic dose and APP for natural causes of death. A crude stratification of APP by dose indicated that APP in patients receiving high dose treatment [for both %BNF (HR: 0.6, 95% CI: 0.4–0.9) and olanzapine equivalence (HR: 0.6, 0.4–0.8)] was associated with a lower risk for natural causes of death. However, this association was not maintained in the fully adjusted models, either for %BNF (HR: 1.4, 0.8–2.3) or olanzapine equivalence (HR: 1.1, 0.8–1.0) as dose measures. We further included %BNF and olanzapine equivalence dose as covariates in two separate models. Similar to the models for all‐cause mortality, the %BNF dose adjustment revealed a modest significant association between APP prescribing and natural causes of death, this association was not significant in the model where olanzapine equivalence was included as a covariate.

Table 3 also summarises the crude and fully adjusted competing risk regression analysis for unnatural causes of death and long‐term APP. We found no evidence to suggest that long‐term APP prescribing was associated with a change in risk for unnatural causes of death. Percentage BNF and olanzapine equivalence dose were included, sequentially in the fully adjusted model. The results indicated that dose had little effect on the overall association.

Table 4 summarises the crude and fully adjusted Cox regression analyses between antipsychotic dose and the risk for all‐cause mortality. We found no association between dose and the risk of death for patients with SMI. The results were very similar for both %BNF and olanzapine equivalence dose definitions.

**Table 4:** Multivariable Cox analysis of the association between all‐cause mortality and antipsychotic polypharmacy dose

Dose calculated as %BNF^a^	HR (95% CI)	*P* value
Unadjusted model	1.0 (0.997–1.000)	*P* = 0.064
Fully adjusted model^b^	1.0 (0.999–1.001)	*P* = 0.996

Dose calculated as olanzapine equivalence	HR (95% CI)	*P* value
1–10 mg	Reference group	
11–20 mg		
Unadjusted model	**0.8 (0.7–0.9)**	***P*** ** = 0.018**
Fully adjusted model^b^	0.9 (0.8–1.1)	*P* = 0.532
21 + mg		
Unadjusted model	1.1 (0.9–1.3)	*P* = 0.296
Fully adjusted model^b^	1.1 (0.9–1.3)	*P* = 0.377

Bold indicates statistically significant value (*P* < 0.05).

a %BNF used as continuous variable.

b Factors included: age, gender, ethnicity, relationship status, employment, deprivation level, comorbid depression (ICD‐10: F32‐33), personality disorder (ICD‐10: F60‐61) and substance use (ICD‐10: F10‐16), time known to SLAM services (days) and smoking.

## Discussion

To our knowledge, this is the first study to investigate the association between regular long‐term APP use (as opposed to APP which is due to pro re nata, cross‐titration or switching) and all‐cause and cause‐specific mortality, taking advantage of a large and diverse cohort and adjusting for multiple confounders, in addition to investigating the effects of combined antipsychotic dose. We hypothesised that as compared to long‐term antipsychotic monotherapy use, long‐term APP would be associated with an increased risk for all‐cause mortality and specifically of death from natural causes. The results indicated a weak association between long‐term antipsychotic polypharmacy use with all‐cause mortality and with natural causes of death, after adjusting for gender and age. Although these associations were not markedly confounded by other factors, the fully adjusted hazard ratios fell below statistical significance. There was no significant association between APP and unnatural causes of death. Also, there was no evidence that antipsychotic dose had a direct effect on the risk of death in this sample with SMI.

In keeping with existing literature, we found that patients prescribed APP were younger, less likely to be employed, less likely to be in a relationship, had a higher proportion of schizophrenia diagnosis and were known to services for longer, in comparison with patients on monotherapy 27, 28, 29. However, apart from gender and age, the aforementioned factors seemed to have small effects on the association between long‐term APP and all‐cause mortality.

Overall, the literature to date examining APP and the risk of death in SMI has been mixed and inconclusive. There has been some evidence from research investigating APP of unspecified duration, indicating that APP increases the risk for death 5, 30. However, findings from larger epidemiological studies have been mixed, with evidence to indicate no association 12 and possibly lower risk for mortality 11 in patients prescribed APP compared to those on monotherapy. Our findings further indicate that the risk in the SMI cohort we examined is not clear‐cut. There did appear to be a small effect of long‐term APP on all‐cause mortality based on the effect estimates, which remained consistent across most models; however, this association was relatively weak and did not reach statistical significance in the fully adjusted model.

People with schizophrenia have an increased risk for premature death from natural causes such as cardiovascular diseases 31, 32, 33 and unnatural causes such as suicide 34, 35 compared with the general population. Risks arising from pharmacotherapy are an obvious concern, particularly when pharmacotherapy regimens are outside standard guidance; however, research examining the effect of long‐term APP prescribing on cause‐specific mortality has been extremely sparse. Bandura and colleagues 10 examined antipsychotic polypharmacy in the ninety days prior to death and reported that the risk for natural causes of death did not increase when patients were prescribed two or more antipsychotics, as compared with monotherapy. In our study, although the findings did not indicate a statistically significant difference between patients prescribed APP and monotherapy in most models, the modest effect estimate for the relationship between APP on the risk for natural causes of death remained consistent, once we adjusted for gender and age. Associations between long‐term APP and unnatural cause of death were weaker and not statistically significant.

In line with existing literature 13, 36, 37, patients prescribed long‐term APP were more likely to be prescribed a higher combined dose of antipsychotics in comparison with patients on long‐term monotherapy. Adjusting for dose had little effect on the association between APP and mortality apart from a small change after adjusting for %BNF. Some previous research has indicated that high antipsychotic dose is associated with increased risk for all‐cause mortality, and more specifically for cancer, cardiovascular and respiratory causes of death 7, 32, 38. There are several possible explanations for discrepancies between studies. It is possible that different methods of calculating antipsychotic dose would yield slightly different results. Methods such as %BNF dose and defined daily dose (DDD) are calculated using the upper licensed dose range of antipsychotics 21. This poses a problem for antipsychotics that reach their maximum efficacy at a lower dose range, such as risperidone (e.g. 3–6 mg a day when the maximum is 16 mg, or quetiapine widely used at 600 mg when the maximum is 750 mg), and which are thus rarely prescribed at maximum or above maximum recommended dose. Furthermore, over the years, there have been changes to the recommended maximum doses for some antipsychotics that make it difficult to compare findings from different studies across time. Therefore, it is possible that this approach offers a less robust measure of dose in comparison with the olanzapine equivalence method. An alternative explanation for differences in findings across studies is the possibility of residual confounding. For example, although Torniainen et al. 7 used age and gender‐matched case and controls, their Cox model did not account for any other factors that may affect mortality such as smoking, which is associated with significant risk for death 24, 39.

This study had several strengths. SLAM is a near‐monopoly mental health care provider for its geographic catchment 15, 16; therefore, we were able to capture a large cohort of patients with SMI giving us the statistical power to adjust for a number of potential confounders, such as smoking and antipsychotic dose, that other research has been unable to examine. At present, there is no ‘gold standard’ of calculating equivalent doses 21; therefore we chose to use two different methods. This gave us an opportunity to test the effect of dose more rigorously and also demonstrate that existing evidence in this field needs to be interpreted with caution, as findings are dependent on the method that is used.

There are several potential limitations that need to be borne in mind when drawing conclusions. It is possible that we did not have sufficient statistical power to detect a consistently significant effect across all models. Furthermore, despite adjusting for multiple confounders, it is possible that some residual confounding may have occurred. For example, we were unable to measure and adjust for the duration of mental disorder. In addition, we were unable to determine the antipsychotic regimen just prior to death or any changes in prescribing in the lead up to patient's death. Consequently, we could not account for the effect of the duration of the prescribed regimen on death. In addition, we were unable to examine the acute effects of antipsychotic regimens. Although this could have resulted in immortal time bias, as the patients had to survive long enough to enter either of the exposure groups, we took the following measures the follow‐up time for both exposure and control group commenced at the point patients were receiving APP or monotherapy for six or more months, therefore both groups entered the cohort after the six months mark in their treatment. In relation to confounders, the role of smoking as a covariate does need to be considered with some caution. Adjustment for smoking presupposes a situation where people who go on to receive APP have more unhealthy lifestyles, including smoking, which account for any raised mortality in this group. However, it is possible that an effect of APP may be to maintain smoking behaviour, if this is used to counteract perceived or actual adverse effects of medication 40. Inclusion of smoking as a covariate in this circumstance would represent an overadjustment. Unfortunately, it was not possible to tease out the timing of APP in relation to smoking status, and therefore these different pathways have yet to be distinguished. Furthermore, examining specific combinations of antipsychotics was beyond the scope of the study.

To conclude our findings suggest that the effect of long‐term APP on mortality is not a clear‐cut one. This has potential implications for further research. It is possible that if there is an effect on mortality, this is driven by specific antipsychotic medication combinations. Therefore, future research could focus on examining common antipsychotic combinations and their effect on particular cause of death, such as cardiovascular death 41. Furthermore, our findings need to be interpreted within the wider clinical and treatment context. Despite the lack of a consistent significant effect of APP on mortality, when prescribing this regimen it is important to bear in mind that APP continues to be associated with more severe side‐effects 42, 43. In addition, the notion that more is better, in relation to adding additional antipsychotics and increasing treatment dose, has been consistently rejected by empirical research 44, indicating that once an optimal dose and response is reached, adding additional treatments makes little difference. Lastly, evidence remains that APP is often prescribed in favour to clozapine monotherapy, despite research indicating that clozapine is effective in treating treatment‐resistant symptoms 45. This is likely to reflect a prescribing culture rather than evidence‐based treatment 45 and a need to target this on prescriber and service level remains 46, 47.

## Role of funding source

This work was supported by the Clinical Records Interactive Search (CRIS) system funded and developed by the National Institute for Health Research (NIHR) Mental Health Biomedical Research Centre at South London and Maudsley NHS Foundation Trust and King's College London and a joint infrastructure grant from Guy's and St Thomas' Charity and the Maudsley Charity (grant number BRC‐2011‐10035). RDH was funded by a Medical Research Council (MRC) Population Health Scientist Fellowship (grant number MR/J01219X/1). RS, HS, C‐KC, RDH and GK receive salary support from the National Institute for Health Research (NIHR) Mental Health Biomedical Research Centre at South London and Maudsley NHS Foundation Trust and King's College London. The views expressed are those of the author(s) and not necessarily those of the NHS, the NIHR or the Department of Health.

## Conflict of interest

RDH, C‐KC, HS and RS have received research funding from Roche, Pfizer, Janssen and Lundbeck. DT has received research funding from BMS, Janssen and Lundbeck. DT is an Advisory Board member in Lundbeck, Servier and Sunovion.
